# An intersectionality framework for identifying relevant covariates in health equity research

**DOI:** 10.3389/fpubh.2024.1286121

**Published:** 2024-03-14

**Authors:** Andrew Simkus, Kristen D. Holtz, Eric C. Twombly

**Affiliations:** KDH Research and Communication, Atlanta, GA, United States

**Keywords:** health equity, intersectionality, framework, impact evaluation, covariates

## Abstract

**Introduction:**

Health equity research uses impact evaluations to estimate the effectiveness of new interventions that aim to mitigate health inequities. Health inequities are influenced by many experiential factors and failure of research to account for such experiential factors and their potential interactions may jeopardize findings and lead to promoted methods that may unintentionally sustain or even worsen the targeted health inequity. Thus, it is imperative that health equity impact evaluations identify and include variables related to the circumstances, conditions, and experiences of the sample being studied in analyses. In this review, we promote intersectionality as a conceptual framework for brainstorming important yet often overlooked covariates in health equity related impact evaluations.

**Methods:**

We briefly review and define concepts and terminology relevant to health equity, then detail four domains of experiential factors that often intersect in ways that may obscure findings: Biological, Social, Environmental, and Economic.

**Results:**

We provide examples of the framework’s application to lupus-related research and examples of covariates used in our own health equity impact evaluations with minority patients who have lupus.

**Discussion:**

Applying an intersectionality framework during covariate selection is an important component to actualizing precision prevention. While we do not provide an exhaustive list, our aim is to provide a springboard for brainstorming meaningful covariates for health equity evaluation that may further help unveil sustainable solutions to persisting health inequities.

## 1 Background

In general, health equity research seeks to understand the causes of disparities in health and healthcare outcomes to identify methods of mitigating them. As such, health equity research largely relies on impact evaluations to demonstrate the effectiveness of new interventions on specified health outcomes related to the targeted inequity. However, health inequities are affected by a barrage of environmental, economic, biological, and sociocultural conditions. Failure to account for such influential conditions and how they may interact in health research may lead to biased findings and promoted methods that can not only fail to mitigate but potentially even worsen the targeted health inequity. Thus, selecting and including variables that are relevant to the targeted health inequity is imperative for tracking the efficacy of a proposed intervention and untangling associations with personal characteristics such as race, which may be related to findings through less overt characteristics such as socioeconomic status, language, income, or differences in disease affliction.

In this paper, we promote intersectionality as a conceptual framework for brainstorming important yet often overlooked covariates in health equity research impact evaluations. Our aim is to present an intersectional health equity framework that describes the confluence of factors related to health outcomes and includes a focus on the personal experiences of individuals with their health condition. We begin by broadly defining health equity and relevant terminology used in health research, as these terms help describe the trajectory and current position of health equity research and the mechanisms by which health inequities persist. Next, we detail four domains of experiential conditions that represent intersecting circumstances that may affect health equity impact evaluations. Finally, we present examples of the framework’s application to lupus-related research and provide examples of covariates used in our own health equity impact evaluations with minority lupus patients as a jumping point for brainstorming meaningful covariates to enrich health equity exploration and intervention.

## 2 Trajectory of health equity terminology

Health disparities: the Centers for Disease Control and Prevention (CDC) defines Health disparities as “*preventable differences in the burden of disease, injury, violence, or opportunities to achieve optimal health that are experienced by socially disadvantaged populations*.” (1) The terms disparities and inequities are often wrongly used interchangeably. Disparities always describe differences between groups; however, disparities are not always inequities. Equal does not necessarily mean equitable, nor does inequal equate with inequity. As noted by Meghani and Gallagher (2), if there are differences in the needs of different groups of people then providing equal services or treatments across these groups would result in inequity (2). In such examples, a disparity in need necessitates a disparity in response to achieve equity. From this conception, disparities are not inherently negative unless they lead to inequity.

Health inequities: are avoidable differences in health that occur between subgroups of the population, both on a national level and between countries. The effects of certain social and economic experiences have the potential to increase the risk of illness and create barriers to receiving appropriate care (3). Health inequities arise for a multitude of reasons; one of the first contributors to health inequities that gained traction in research was the concept of health literacy. The CDC defines personal health literacy as “*the degree to which individuals have the ability to find, understand, and use information and services to inform health-related decisions and actions for themselves and others* (4).”

Health equity: the CDC defines Health equity as “*the state in which everyone has a fair and just opportunity to attain their highest level of health* (5).” For many people, there are economic and social barriers to health and healthcare that require systematic action to mitigate. Disparities in health occur when certain groups disproportionately experience a certain condition or illness (6). Health equity exists when disparities in health-related outcomes are unable to be predicted by economic, environmental, or sociocultural conditions. Health equity is an intersectional concept since individuals simultaneously belong to a multitude of groups and thus may experience overlapping inequalities. This intersectionality also has the potential to obscure existing inequities if research fails to account for relevant factors in the analyses.

Initially, health research had to prove the existence of disparities and therefore much research has focused on identifying and defining health disparities and the particular groups most affected by them. However, by now it has been well established that health disparities and inequities exist and persist among certain subgroups and settings. This being the case, it has been argued that health research should shift the framing narrative from being problem-oriented to being solution-oriented; from a disparity-based model to an equity-based model (7). Health disparities is a more deficit-based framework (what is different or lacking) while health equity is more aspirational/strengths oriented in approach. By moving from identifying the problems to identifying the solutions, can we assess what measures are required to achieve health equity.

## 3 Intersectional experiential conditions

Social determinants of health are the circumstances people are born into, develop in, live in, and work in, which often vary in accessibility and ability to address illness. Such circumstances are affected by broad systemic issues including socioeconomics, development, and even local policies (8). In 2008, the World Health Organization (WHO) concluded that social determinants of health are mostly responsible for inequalities in health and healthcare worldwide (9). Srinivasan and Williams (7) argue that in the United States (US) racial and ethnic framing of health disparities has largely ignored impactful socioeconomic factors which are often associated with race and ethnicity (7). Socioeconomic status is primarily characterized by employment, education, and income level (10). Individuals with lower socioeconomic status are at substantially higher risk of experiencing worse health outcomes, a phenomenon commonly known as the social gradient (10). Below, we briefly explore circumstances that may affect health equity and highlight the intersectional nature of these conditions.

Intersectionality as a theoretical framework proposes that multiple experiential conditions intersect at individual, community, and structural levels, disproportionately creating barriers and privileges among groups and individuals (11). Intersectionality has been used to construct assistive frameworks for health equity research, such as the Health Equity Framework (HEF) developed by Education, Training and Research (ETR) (5), which served as the main inspiration in our own framework/approach for covariate selection.

Similar to the HEF, we explore four key domains of intersecting experiential conditions known to directly affect health outcomes worldwide; however, our categorizations are specifically broken down into categories which may be more practical for helping identify relevant covariates for health-related impact evaluation: (1) biological, (2) social, (3) environmental, and (4) economic. While we list these experiential conditions categorically, these are intersectional conditions which often overlap or interact with one another to create unique experiences for different subgroups of the population. This intersectionality can also help identify beneficial or protective factors, an important component of any health equity-based model.

Interactions that often occur between these co-variates are complex in nature, driven by obscure and often debated mechanisms. Increasing evidence of gene–environment interactions has proven that some genetic risk factors previously considered to be unalterable only present themselves alongside certain social or environmental triggers which are alterable. Such triggers are important to unveil and offer areas of key importance for social protection policy. As Braveman et al. (12) assert, we need rigorous scientific standards for building evidence around such interactions that can help guide social protective policies that consider the impact from upstream determinants of health over time (12, 13).

### 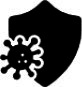 Biological conditions

3.1

Biological factors include unchangeable genetic conditions such as age, sex, race/ethnicity, and other inherited factors such as disease, body type, brain chemistry, hormonal levels, nutrition, and sometimes even psychological characteristics (14). A multitude of diseases have been found to infect and affect individuals of differing racial/ethnic backgrounds differently and often minorities experience the highest risk of disease-related morbidity and mortality (15). There are a wide range of genetic predispositions that increase the likelihood that an individual experiences certain illnesses or conditions. Genetics can also impact modifiable health behaviors which may mitigate or trigger further health risk. Level of physical activity for example, has been found to vary depending on family genetics, age, sex, race/ethnicity, pathology, and body mass index (12). Research suggests that differences in the pleasure and reward systems within the brain may contribute to differences in exercise adherence (16). In such scenarios, further motivation, or intervention to increase exercise levels could have substantial impacts on wellbeing.

Studies assessing the association between genetics and health outcomes among monozygotic twins with nearly identical genotypes have shown that increased physical exercise often helps mitigate inherited health risks (17, 18). Furthermore, illness may have serious consequences on an individual’s experiences and abilities. While research has confirmed the presence of unchangeable biological differences in health predispositions and outcomes among certain races and ethnicities, there are many economic, environmental, and social factors that are strongly associated with race/ethnicity which may overlap in ways that create barriers to care. These changeable conditions/disparities that create barriers to care reveal avenues for solution-oriented, equity-based intervention.

### 
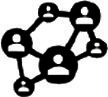
 Sociocultural conditions

3.2

Social cohesion/integration, discrimination, stress, health behaviors, community engagement, and support systems all contribute to health outcomes (19). Social conditions such as stress are arguably the most overlapping theme across these categories. Biological, economic, sociocultural, and environmental differences can all interact with an individual’s coping mechanisms and stress levels (20). High levels of stress have been proven to be harmful to the immune system and can actually aggravate or trigger a host of diseases and psychological disorders (21). Individuals exposed to stressful living or working environments are known to experience higher rates of illness and mental health disorders (22, 23). Culture relates to the ways that individuals experience and express social experiences, including beliefs about illness and how to respond to illness (24). Research has shown that risk communication and educational materials are most efficacious when they are tailored to the culture of the group being targeted (25).

### 
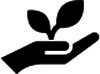
 Environmental conditions

3.3

At its most basic level, one’s environment begins with the physical infrastructure of their personal home and that of their surrounding community. In the US, the leading causes of disease, disability, and preventable deaths are strongly correlated with conditions inside the home (26). For example, disproportionate rates of asthma, lead poisoning, falls, burns, drownings, and radon related cancers are each strongly related to inadequate household conditions (26). A multitude of inadequate housing conditions contribute to health adversities including physical, chemical, biological, and building-related factors (26–28).

### 
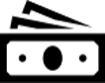
 Economic conditions

3.4

There is irrefutable evidence that socioeconomic status contributes majorly to trends witnessed in health-related autonomy, morbidity, and mortality (29). In regard to health outcomes, prior research has identified significant interaction effects between both race and educational level and race and employment status (29). Economic conditions are known to have differing effects on individuals depending on biological factors. For example, Farmer and Ferraro (29) found that White adults were more likely than Black adults to experience increases in self-reported health as their education level increased (29). Profession is a strong predictor of health-related behavior for males (30), and occupational risks and safety standards may also directly affect overall health and disability (12). Furthermore, economic conditions directly relate to the quality and safety of one’s living environment.

While each of these covariate categories are directly related to health outcomes, by exploring these covariate categories together, the intersectional nature between them becomes more apparent.

## 4 Intersectionality of conditions

While on the surface it is tidy to break these social determinants into separate classifications, there is no ignoring the overlap and interaction effects that occur between many social determinants of health. Economic conditions, for example, interact with most if not all social determinants. Those with better jobs are more likely to make more money, those who make more money can afford to live in safer neighborhoods and engage in healthier behaviors. Obviously, economic barriers to protective factors like healthcare services, insurance, healthy environments, and nutrition also have direct effects on health outcomes. Worldwide, in low-, middle- and high-income countries the poorest individuals are known to experience the worst health outcomes, a concept known as the social gradient. On average, the lower an individual’s socioeconomic position the worse their health compares to others in their country (8).

Many of the environmental, social, and economic conditions we have discussed are associated with health outcomes through barriers to care. Hong et al. (31), found that barriers to care were equally important as common demographics in explaining variance in self-reported health (31). Common barriers to care include a lack of knowledge among healthcare providers regarding a certain condition or treatment (32, 33), a lack of patient awareness about services available (34), absence of insurance (34), transportation or mobility issues (35), language limitations (36), and even perceptions of trust in healthcare institutions (34). Each of these barriers may easily be seen as relating to socioeconomic status (SES) (37) and indirectly to race/ethnicity. Under a health equity lens, these barriers also represent inverse protective factors that may assist in identifying pertinent interventions. For example, specifically tailored educational interventions can address knowledge and awareness gaps among both patients and healthcare providers.

## 5 Application of the intersectional framework to an understanding of systemic lupus erythematosus

Systemic lupus erythematosus (SLE) is an inflammatory autoimmune disease that disproportionately affects young minority females worldwide (38). Differences in the incidence and prevalence rates of SLE have been observed by sex, race/ethnicity, location, and time (39). Worldwide, SLE is more common among females across all ages and ethnic groups. Black individuals have the highest SLE incidence and prevalence rates while White individuals have the lowest. Furthermore, compared to non-Hispanic White patients with SLE, Black and Latino patients with SLE experience increased severity of disease symptoms, more frequent lupus-related complications, and up to three-times higher mortality rates (40–42).

The highest SLE incidence and prevalence rates have been estimated within North America at 23.2/100,000 person-years and 241/100,000 individuals. SLE is more commonly found in urban areas than rural areas (43). In the US, Black patients account for approximately 43% of SLE diagnoses, yet only comprise 14% of lupus-related randomized clinical trials (RCTs) (42). In comparison, White patients account for 33% of prevalent SLE diagnoses while comprising 51% of lupus-related RCT representation (42). The Food and Drug Administration (FDA) requires randomized clinical trials (RCTs) on new medical treatments to assess their safety and efficacy among all subgroups of the population. An individual’s genetics can affect their predisposition to illness and also their body’s response to treatment (44). Underrepresentation of minority groups in RCTs makes it difficult for the FDA to approve new treatments for use with minority patients, potentially leading to further health disparities (45). Lupus-related interventions often attempt to understand and address these disparities in RCT participation.

An intersectional framework fortifies intervention planning and evaluation by maximizing the consideration of influential factors and conditions which relate to patient outcomes such as RCT participation. This gestalt approach pushes researchers to identify and test more discrete influential factors that may intersect with variables found to be significant in prior research, yielding new avenues for targeted intervention. While an intersectionality framework can seem overwhelming with more variables and potential interactions to explore, the overall goal is to gain precision in identifying which modifiable actions lead to the best outcomes among differing conditions.

Below, we list examples of potential covariates relevant to lupus-related health equity impact evaluation studies. We tie each of these covariates to the four domains of experiential conditions detailed above and explore how these conditions may potentially intersect to create either detrimental or protective experiences for patients with SLE.

### 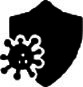 Biological

5.1

- Race/ethnicity: Black and Latino individuals experience disproportionately higher prevalence of, more severe manifestations of, and higher morbidity from SLE compared to other groups (46). When assessing race and ethnicity as covariates, researchers often use both variables as separate covariates, however merging race and ethnicity variables may yield further insights when sample size is large enough. The US census bureau found in 2016 that by adding a full list of combined race and ethnicity answer choices to the survey, Latino respondents were more likely to make use of the standard choices, increasing representation and response quality (47).
- Sex: Women experience nine times the prevalence of SLE compared to men (46). Hormonal exposures and reproductive issues among women have been linked to interactions with genetic risk factors (48).
- Genetics: Having a higher genetic risk of SLE has been linked to earlier onset of and more severe manifestations of the disease (49). Furthermore, genetic predispositions have been found to likely interact with environmental variables to trigger SLE symptoms (49).
- Behavioral risk factors: There are behavioral risk factors that have been found to biologically converge with genetic risk of SLE including, cigarette smoking, previous trauma, obesity, stress, and even lower alcohol intake (48).
- Health status: Individuals with SLE are more likely to experience extreme bouts of fatigue and disability with daily tasks. Additionally, neurological and mental health issues have been associated with significantly worse quality of life outcomes among patients with SLE, particularly anxiety and depression (50). Depression alone has been linked to worsened organ damage due to SLE (51).

### 
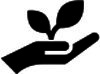
 Sociocultural

5.2

- Social support networks: Research has linked lack of social support to depression, and in turn, to worsened organ damage due to SLE (51). Social networks are a protective factor emotionally and may also assist SLE patients with such things as transportation, food, and daily tasks (52).
- Experiences with discrimination: Perceptions of experienced racism when engaging with the healthcare system for SLE management has been linked to higher depression (53). Research has shown that psychosocial stress is positively associated with SLE flares (54).
- Immigration status: Non-documented migrants may have difficulties accessing health services, limited resources, language barriers, and fears relating to immigration status including the potential for deportation (55). Each of these issues may make immigrants with SLE more vulnerable and less likely to receive treatment.
- Language proficiency: Certain groups of SLE patients are more likely to experience language barriers, which clearly relates to communication struggles between providers and patients. Treatment adherence studies have shown that SLE patients sometimes report language barriers as reasons for not adhering to their treatment plan (56).
- Healthcare access: Access to healthcare is the most important determinant of whether SLE patients receive the treatment they require. This access may be obscured by several factors including whether an individual lives in a rural or urban area, insurance status, transportation ability, awareness of services, language barriers, perceptions of trust, etc.
- Cultural beliefs/practices: Some cultures may have less trust in western medicine, and may rely more on culturally traditional herbal medicines, or may have religious beliefs against seeking medical treatment.
- Risk communication: Communication is relevant to each of these aforementioned points. A provider or treatment team member’s ability to effectively communicate risks associated with SLE and the relevant available treatments and clinical trials may impact a patient’s ability to identify efficacious treatment and lifestyle choices that may benefit their condition. Providers are most often the source of their patients’ information about potential clinical trials and treatment (57); however, providers often lack access to clinical trial information or familiarity with referral processes and clinical trial site locations (58). Because of this, many interventions for increasing knowledge about SLE and available treatments has taken the form of patient advocacy and provider education (59, 60).

### 
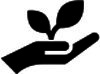
 Environmental

5.3

- Urban/rural residence: The area that SLE patients live in can drastically affect not only their access to healthcare but the likelihood of early diagnosis, and the severity of their experience with the disease. Urban SLE patients are diagnosed much earlier on average, while rural patients are more likely to experience moderate-to-severe manifestations of SLE, and to present with symptoms of oral ulcers, malar rash, photosensitivity, and arthritis (61). SLE patients in rural areas are known to experience higher rates of disease activity, kidney disease, musculoskeletal complications, and depression, likely due to poorer access to healthcare (62).
- Pollution levels: Environmental factors, including air pollution and environmental silica have been linked to interactions with genetic risk factors as a contribution to SLE (48).
- Neighborhood walkability: Community factors can determine behavioral risk factors like the ones we explored above. The more walkable a neighborhood the more likely the community is to get exercise. The more exercise patients with SLE get, the less likely they are to experience problems with weight, heart disease, and disability (63).

### 
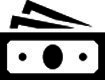
 Economic

5.4

- Income: Having a lower income obviously relates to many, if not most health-relevant factors including housing conditions/environment, education, nutrition, type of work, etc. Among SLE patients, lower income has been linked to higher likelihood of organ damage and lower rates of survival (64, 65).
- Education: While education is often viewed as a protective factor, the impact of education on health outcomes is known to vary among SLE patients. For instance, higher education levels have been linked to lower mortality among White SLE patients, but not for minority SLE patients (66). Education is also related to the likelihood of being diagnosed with SLE in the first place, which may help explain why a mediating effect has not been observed among poorer minorities who tend to have lower education attainment (66).
- Employment status: SLE often causes cognitive impairment and memory issues, severe lupus-related memory issues have been liked to employment status (67).
- Type of occupation: Disease activity, flares, and organ damage may make physically demanding jobs quite difficult for patients with SLE (68). Fatigue and other psychiatric and neurological disorders that often cooccur with SLE such as depression may also severely limit the types of work related tasks patients with SLE are able to perform (50).
- Nutrition: A balanced diet has been shown to have a protective effect on SLE patients’ abilities to prevent and manage lupus-related symptoms, contributing to reduced disease activity and lower likelihood of co-morbidities (69). Appropriate dieting among SLE patients has been associated with longer periods of disease remission and may also mitigate adverse effects from medications experienced from medication (70).

## 6 Examples for covariate selection

We often employ regression modeling to consider the effects of confounders on the strength of our health equity interventions. Because the choice of confounders included in the model can reveal insightful circumstances for future interventions it is important to gather as much data as seems reasonable from participants on their personal experiences. While classic covariates in health equity research include race, sex, age, and income we argue the need to delve deeper into the participant’s intersecting experiences and circumstances. Below, we provide examples of potential covariates for research specifically targeting minority patients who have SLE; however, we believe these to be relevant and applicable to a wide range of health conditions and health equity-related intervention. In depth qualitative research that engages and details patients’ personal experiences provides excellent opportunities to identify which covariates may be insightful to control for in a given study. By including these experiences in analyses, we may better understand both the hurdles and facilitators of intervention success.

### 6.1 Patient experience with SLE itself

Examples: Attempts to quantify a patient’s experience with SLE may wish to assess how long the patient has been diagnosed, how many flare ups the patient experienced in the past year, how many doctor visits and/or emergency room visits they had in the past year. Disease activity scores may also be meaningful to evaluate.

### 6.2 Level of social support

Examples: Likert-type scales may assess the degree to which the patient feels they have adequate support networks emotionally and physically, including family, friends, or professional services actively engaged in the patient’s life.

### 6.3 Provider stability/training

Examples: Likert-type scales may assess patient satisfaction with management of their healthcare and trust in their providers’ ability to help them manage their condition.

### 6.4 Quality of life

Past month frequency of mental and physical health struggles the patient may experience and the frequency that these struggles impact the patient’s ability to manage self-care, sustain employment, and engage in activities they find meaningful.

## 7 Employing intersectionality in health equity impact evaluation

The intersecting nature of health-related disparities is exemplified in a number of ways with SLE, making SLE ripe ground for health equity-based interventions. (1) There are genetic differences in prevalence rates of SLE (46, 49), (2) there are impacts of SES levels on groups with prevalent SLE that may affect access to care and representation in RCTs (37), (3) lower representation in RCTs equates to more difficulty identifying efficacious treatments (45), (4) historical and institutional racism creates further barriers and divides of distrust among groups with prevalent SLE (71), and (5) lack of efficacious treatments for minority SLE patients likely equates to lower work ability, and resulting economic differences may influence behavioral, social, and environmental factors that are known to further affect one’s experience with SLE.

A health equity lens views these intersecting disparities as actionable opportunities for intervention. A comprehensive approach to achieving health equity would effectively target multiple angles of these health-related disparities simultaneously. From an educational perspective, training courses and peer interventions can inform patients, community workers, and medical professionals about the disproportionate affliction of SLE across race and sex, and the importance of seeking relevant care (59, 60).

Targeted risk communication and educational materials that are specifically tailored to the communities with highest risk are likely to be most efficacious at improving patient engagement with the healthcare system (25). Educational materials can also provide tools for diagnosing symptoms, bolstering referrals to relevant RCTs, and enhancing skills in building rapport with communities that may historically lack trust in the medical system (59, 60). From a medical perspective, increasing minority involvement in RCTs should assist in identifying efficacious treatments for minority SLE patients. Identifying effective treatments will likely increase the capability of minority SLE patients to sustain full-time work and avoid economic struggle. SLE patients themselves, due to their lived experience, may be considered experts regarding types of supports that are most beneficial, and therefore, should be included in efforts to guide public health initiatives for early diagnosis and disease management.

As Liang et al. (72) specified, we can improve access to high-quality health care, promote understanding and awareness of SLE, and the conditions that may affect lupus-related outcomes. We can develop support programs to assist with self-monitoring and tracking adherence to treatment-related protocols. And, we can increase community efforts to create opportunities that facilitate tasks that may be overwhelming to patients with SLE such as childrearing, homemaking, transportation, and employment (72).

### 7.1 Efficiency of intersectionality

Considering the intersectional nature of relevant covariates allows research to explore how variables historically associated with certain subgroups of the population may be better ascribed to the experiential conditions these groups are likely to encounter. As research progresses in identifying the intersecting experiences and conditions responsible for health disparities, interventions and policies will be better informed about how to adequately meet the needs of individuals facing these disparities. Thus, intersectionality applies both to understanding the problem and to finding efficacious and sustainable solutions. Through well targeted health equity-based intervention, multiple disparities may be addressed simultaneously.

## 8 Conclusion

As we have explored, the intersectional nature of life experiences and disease may obscure, hinder, or facilitate the success of an intervention. In this paper, we explore how intersectionality applies to health equity-based intervention and provide examples particularly related to SLE on how these intersecting experiences and conditions can create detrimental and/or protective factors for the patients experiencing them. Our intersectionality framework for covariate selection is meant as a general steppingstone; however, these insights may be limited by our experiences focusing on health equity impact evaluations among patients with SLE. While we explore potential covariates relevant to a wide array of health equity evaluation, we hope that these examples can inspire other focuses in health equity research to explore, identify, and promote the interactions found to impact health outcomes, and when/where protective factors can be most impactful.

Another limitation with an intersectionality approach of brainstorming and maximizing related covariates is concern regarding multicollinearity (73, 74). Covariates that predict SLE outcomes may also be highly correlated with each other, creating problems for estimation. Methods such as testing the variance inflation factor (VIF) (75) are helpful in assessing how much of the variance in a coefficient estimate’s is inflated due to multicollinearity. Exploring multiple sets of regression models and potential interaction terms will assist in identifying main effects and interaction. Researchers may also assess categorical differences associated with key variables by use of generalized linear mixed models (74). Depending on the types of data being used, appropriate analytical methods and rigorous consideration of intersectional relationships will strengthen final estimations.

While not an exhaustive list, we believe the covariate examples we provide in this paper may be helpful for inspiring meaningful covariate selection across other fields of health equity-based impact evaluation. A health equity framework is important for building solution-oriented interventions; understanding both positive and negative factors can help develop interventional support that is optimally impactful. While considering the intersectionality of health-related covariates can be challenging, it provides the most nuanced option for creating real, lasting change. Moving beyond demographic variables as our predictors and controls will make our work as public health interventionists real life.

## Author contributions

AS: Conceptualization, Writing – original draft, Writing – review & editing. KH: Project administration, Writing – review & editing. ET: Project administration, Supervision.
